# Who is the Scientist-Subject? A Critique of the Neo-Kantian Scientist-Subject in Lorraine Daston and Peter Galison’s *Objectivity*

**DOI:** 10.1007/s11024-017-9313-5

**Published:** 2017-01-30

**Authors:** Esha Shah

**Affiliations:** Water Resources Management Group, Department of Environmental Sciences, Wageningen University, Drovendaalsesteeg 3a, 6708 PB Wageningen, The Netherlands

**Keywords:** Subjectivity in science, Neo-Kantian scientist-self, Co-implication of psychology and epistemology, Role of affect in science, Lorraine Daston, History of subjectivity

## Abstract

The main focus of this essay is to closely engage with the role of scientist-subjectivity in the making of objectivity in Lorraine Daston and Peter Galison’s book *Objectivity,* and Daston’s later and earlier works *On Scientific Observation* and *The Moral Economy of Science.* I have posited four challenges to the neo-Kantian and Foucauldian constructions of the co-implication of psychology and epistemology presented in these texts. Firstly, following Jacques Lacan’s work, I have argued that the subject of science constituted by the mode of modern science suffers from paranoia. It is not the fear of subjectivity interfering with objectivity but the impossibility of knowing the truth of the *real* that causes paranoia. Here, I have argued that it is not the ethos of objectivity that drives epistemology as Daston and Galison suggest, but the pathos of paranoia. The second challenge builds upon Kant’s own denial that the perfect correspondence between the human will and the moral law is possible. Kant himself thought that an ethical human act is impossible without the component of “pathology.” This questions Daston and Galison’s argument that there is always ethical imperative at the core of epistemic virtue. The third challenge contests the way Daston and Galison take *appearance for being* in their application of the Foucauldian concept of *technologies of the self* in modeling the master scientist-self. The fourth challenge questions the notion of the psychological and unconscious in the making of epistemology in Daston’s later and earlier work. Against this background, I aim to make a claim that understanding and disclosing “entities” in the scientific domain presupposes an understanding of “being” in general. My goal is to open up the discussion for an alternative conception of the scientist-subject and thereby an affective and existential formulation of science.

My parents, grandparents, great-grandparents, my brothers and sisters, and all my family, and those close to me, people who are my friends, those intimately near to me, as well as my comrades and my acquaintances, together with all those more distant people who, during these last years, have been around me, that is to say army people, employees, my financial and worldly relations, have most often deceived, insulted, scorned, railed, mocked, mumbled, dishonoured, brutalised, thrashed me….^1^

These were the words of the well-known mathematician Georg Cantor who often suffered psychotic episodes and was hospitalized many times during the development of his revolutionary set theories – now known as Cantorian set theories foundationally important in mathematics and science (Burgoyne 2002: 237). Psychoanalyst Imre Hermann, who studied the creative work of Cantor, argued that this was actually an example of parallelism between the psychic structure of Cantor’s manic-depressive episodes and the structure of his set theory.^2^ If one closely looks at the structure of the statement, there are two types of entities here – the people and Cantor’s perception of what these people are doing to him. Cantor’s relationship with the first entity becomes distant as the list progresses – begins with parents and ends with the general worldly relations. But in the list of the second entity, Cantor’s perception of these people’s actions become more and more physically intense; beginning with being deceived, Cantor feels brutalized and thrashed as the list progresses – the stranger the person the more brutalizing the impact. This is the core of the set theory in mathematics: how the entities in one set relate to the entities in the other, especially when both sets encompass the infinity of members.

“What are the psychic pre-requisites for the advance of science? How the analysis of the soul is structured vis-à-vis the analysis of science?” These were the questions Freud was grappling with in his last work *Moses and Monotheism* published in 1938. The same question is asked differently in recent times. “Is Science emotional?” asks White (2009). Such a question about science might even mean a heresy. The view that science, supposed to be objective, depends in essential ways upon highly specific constellations of emotional and existential – subjective – experiences is fundamentally paradoxical. In fact, subjectivity and objectivity are commonly arrayed in opposition. More than often, objectivity is a denial, willful control, or erasure of subjectivity. Steven Shapin calls it a dustbin conception of subjectivity – the bin collects those stories that deflate, disrupt or disorder objectivity (Shapin 2012: 171–172). Not only that subjectivity disrupts objectivity, but the role of individual scientists’ subjectivity in the making of science is often obliterated, treated as merely incidental, designated as mind reading, or relegated to biographies. Even when subjectivity is accounted as co-implicated with objectivity, it is treated only as mental states of collectives. Lorraine Daston discusses the way certain methodological paradigms such as empiricism and objectivity require *moral economies* of *affect-saturated values* that define mental states of collectives (Daston 1995, 2008). In their historical analysis of the co-production of subjectivity-objectivity, Daston and Galison insist that they were concerned with the collectives and not with the individual psychology (Daston and Galison 2007). Steven Shapin also warns against the risk of “atomising particularlism” and suggests that the individual reflexes should be disciplined by sociologists’ collectivism (Shapin 1992: 354–355). And when the scientist’s subjectivity is indeed treated in individual capacity, it is predominantly constructed as the neo-Kantian ideal – a unified and wilful, self-determined, self-regulated, active and autonomous, rational subject wilfully driven by social and scientific ethos. Another approach popular among historians is the scientist-subject as a Foucauldian construct, who is reduced to the effects of power. Cantor is the kind of example that historians of science are more likely to treat as a genius scientist gone mad with whom they would be wary of engaging outside the biographical pages. Taking the example of Cantor, I wish to modify White’s question “Is science emotional?” into “Who is the scientist-subject doing science?” In fact, this question is being increasingly engaged with in the recent works in history of science.^3^ And still, it would be hard to find the “mad excess” of Cantor, especially his delirium and his suffering, treated as not just integral but indispensable part of his science.

The purpose of this essay is to closely engage with the work of Lorraine Daston and her co-authored work with Peter Galison on the history of scientist-subjectivity in the making of objective science. The essay mainly engages with Daston and Galison’s book *Objectivity* (Daston and Galison 2007) while it also discusses Daston’s later work *On Scientific Observation*, and her earlier essay *The Moral Economy of Science* (Daston 1995, 2008). The main purpose of this essay is to posit four challenges to the neo-Kantian and Foucauldian constructions of the co-implication of psychology and epistemology presented in these texts. Challenging Daston and Galison’s argument of the co-construction of psychology/subjectivity and epistemology/objectivity, I want to propose that understanding and disclosing “entities” in scientific domain presupposes an understanding of “being” in general. By critiquing the neo-Kantian scientist-self I wish to make a case for an alternative conception of the scientist-subject and accordingly an affective and existential conception of science. I wish to claim, following Michael Polanyi (following Sartre), that *existence precedes essence*, or in other words, psychology precedes epistemology.

## The Scientist-Self in *Objectivity*

In their book *Objectivity,* Lorraine Daston and Peter Galison discuss a rich array of scientific atlases as records of the co-construction of objectivity-subjectivity. Scientific atlas is a compendium or a catalogue of visual and other images. The authors show how the atlas standardized and recorded ideal practices of both selfhood and objective science. They discuss three different forms of epistemological practices – truth-to-nature, mechanical objectivity, and trained judgment in the making of science represented by these atlases. These epistemological virtues and the corresponding notions of subjectivity, although chronologically unfolded in succession since the 18^th^ to 20^th^ centuries, were not mutually exclusive. Each successive stage built upon as well as reacted to the earlier ones. The main argument of the book is anchored on mechanical objectivity of the mid-19^th^ to early 20^th^ century. Scientists in these atlases are portrayed as self-disciplined, steel-willed, even self-denying individuals, who are motivated by a strong moral drive. The core task of the scientist-self in the second half of the 19^th^ century was to diminish by strength of will the subjective in the making of the objective. This would mean expelling all forms of prejudice, even skill, fantasy, emotional attachment, and judgment in order to make the cognitive and the perceptual not only “verifiable, epistemologically warranted and communicable” but also the “exact” and hence the “objective” replica of nature. The scientist-self corresponding to truth-to-nature objectivity was a sage whereas the one responded to mechanical objectivity was the indefatigable worker, and the one who practiced trained judgment as objectivity was intuitive expert (Daston and Galison 2007: 3–7).

The central claim of Daston and Galison’s *Objectivity* therefore is not only to show that objectivity has history and hence it is variable, but more importantly to demonstrate how objectivity – or, in other words, epistemology – is *always* inter-articulated with ethos and forms of subjectivity. What is important to note here is that the virtue of mechanical objectivity is asserted only by the suppression or expulsion of subjective forces, which is achieved by exercising extreme efforts of will – this willfulness forms the core of the objective scientist-self. In fact, Daston and Galison compare the willfulness with asceticism. They claim that “objectivity is to epistemology what extreme asceticism is to morality”(Daston and Galison 2007: 374). They further clarify that the demands of objectivity compel the knower to develop the most strenuous forms of self-cultivation, which even borders on the brink of self-denial and self-destruction (Daston and Galison 2007: 40). Daston and Galison thus insist on a strong link between epistemology and ethics; they argue that in pursuit of objectivity scientists convert themselves to another style of life like ascetics or religious philosophers of the antiquity. In short, in making a case that the changes in subjectivity are co-implicated with the changes in objectivity, Daston and Galison construct the scientific-self as a neo-Kantian ideal – a determinate, regulated, active, and autonomous subject driven by willful ascetic ethos.

This willful self, however, is acknowledged to constitute fear. In her article *On Scientific Observation,* which followed the publication of *Objectivity,* Daston argues that the fear that the subjective lenses might filter or distort objective empirical results have always remained in the background (Daston 2008: 97–98). Fear as constitutive of the scientist-self has a much stronger place in the book *Objectivity.* “In all cases, it is fear that drives epistemology,” write Daston and Galison (Daston and Galison 2007: 49). All epistemology begins in fear – the fear that “the world is too complex or the human intellect is too weak to grasp this complexity” (Daston and Galison 2007: 49). Objectivity is one such chapter in this history of intellectual fear. Daston and Galison, however, contend that the fear objectivity addresses is different from and deeper than the others. The threat to objectivity is not external, it is rather internal – “*Objectivity fears subjectivity, the core self*” (Daston and Galison 2007: 372–374).

As one reviewer argues, if the fear, not ethos but pathos drive epistemology, then causality is introduced between psychology and epistemology – in the sense that psychology/subjectivity and epistemology/objectivity are no longer co-constructed but psychology precedes by driving epistemology (Anderson 2008: 660–661). A number of reviewers raised similar issues with the book *Objectivity.* For example, one reviewer asked: “Is Objectivity an emanation of more fundamental subjectivity, a subjectivity that is afraid of error, of the inaccuracies that ensue from any act of willfulness, afraid ultimately of itself?” (Switzer 2009: 100–101). Paul White called it an outbreak of “epistemic fear” on the pages of *Victorian Studies* (the journal in which this debate on the book took place) (White 2009: 796).

In their response to the reviewers, Daston and Galison make a case that their conception of the way the knower relates to the knowledge is *orthogonal* (italics mine) to the familiar psychological and sociological ways to understand this. They interpret psychological as pertaining to the knower as an individual with specific emotional and intellectual life trajectory, whereas sociological as relating to the knower as a member of a collective. They theorize their own conception of the fear as a kind of “hybrid monster” that straddles the boundaries between psychological and sociological, and psychological and epistemological. They insist that the fear is genuinely epistemic. It is a “response to genuine and multiple obstacles to the acquisition of knowledge” (Daston and Galison 2008: 671). The fear is an “affective state” not incompatible with epistemology. They say, “epistemology is rooted in an ethos which is at once normative and affective – *or affective because normative*” (Daston and Galison 2008: 671, italics mine). In short, for Daston and Galison, the epistemic fear is psychological, collective, and epistemological at the same time, and hence it is neither pathological nor its occurrence foreground psychology prior to epistemology as one of the reviewers contends. The key aspect of Daston and Galison’s insistence is that the fear is “not irrational,” in fact, it is “reasonable,” they argue. This response would indeed keep intact the neo-Kantian sense of the self. The self that is afraid of itself is so only in the noble pursuit of objectivity. This self, first and foremost, is normative; it is determined to do the right thing; it is affective (fearful) only because it is normative. Active self-consciousness is thus the ground upon which both the ethics of knowledge and knowledge are built.

Below, I aim to posit four challenges to Daston and Galison’s central argument on how epistemology and ethos fuse. I wish to argue that the (epistemic) fear is not rational but “irrational”; it is not ethical but “pathological.” I must point out that both these terms “irrational” and “pathological” have specific meanings that I will clarify and discuss later in the essay. The first challenge is derived from Jacques Lacan’s argument that the subject of science constituted by the mode of modern science suffers from “paranoia.” The second challenge builds upon Kant’s own denial that the perfect correspondence between the moral law and the human will is possible. Kant in fact thought that an ethical human act is impossible without a “pathological” component. The third challenge questions the way Daston and Galison have taken appearance for being in their application of the Foucauldian concept of *technologies of the self* to constructing the scientist-subject of mechanical objectivity. And the fourth challenge contests the notion of the unconscious in especially Daston’s earlier and later work.

### “Impossibility of Knowing the Real Causes Paranoia”

For the first challenge, I want to discuss the example that Daston and Galison open their book with. The British physicist Arthur Worthington is working on the photographic images taken a few thousandths of a second apart of impact caused by a liquid drop. He is working on a compendium of hand-drawn sketches of droplet images since 1875 that eventually contributed to the branch of fluid mechanics. For Worthington, the splash of milk droplet hitting a hard surface or dropping into a liquid break into a “perfect symmetry” (Daston and Galison 2007: 11). He ignores the accidental specificity and peculiarity of what he thinks is a defective splash and rather sets out to capture the world in its “types and regularities.” The retina impressions of perfection and symmetry, however, were shattered once he captured these images for the first time in photographs in 1884. The photographs showed irregularities instead of symmetries. Not only that the imperfection of nature was shocking but what Worthington found disturbing was the realization that he was deceived of idealized, symmetrical mirages for 21 years, he was concerned that even when he did encounter many irregular and unsymmetrical images, in compiling these images for the compendium his mind chose only the “ideal” and symmetrical splashes and rejected irregularities. Worthington concluded that the selection of idealized images was in fact the error of judgment of a fallible human. Daston and Galison argue that Worthington was not alone in rejecting the irregular. The choice of perfect over imperfect was profoundly entrenched in the scientific practices at that time. With the mechanical camera replacing the mind’s eye in 1895, the earlier epistemological virtue of documenting perfect and regular images of nature was reduced to a psychological fault, a defect in perception (Daston and Galison 2007: 13–16).

Worthington’s realization has an important character. The transition from the subjective and hence fallible human judgment to the mechanically-produced, infallible perception meant a major shift in the way nature was visualized. From being symmetrical regularity, the physical world now acquired asymmetrical individuality and as a result became profoundly complex. In Worthington’s terminology the objective view – the new epistemic virtue – was required to capture this “real” as opposed to the subjectively-produced “imaginary” nature. Daston and Galison argue that mechanical objectivity inaugurated new subjectivity that was required to willfully reject all human intervention, all subjective elements, in the representation of nature. In projecting this new objectivity-subjectivity, however, Daston and Galison underplay the shift in the scientist’s attitude towards nature. Objectivity now meant a “faithful,” “real,” “exact,” “as it was” representation of nature. The purpose of objectivity, including the mechanical devices like microscopes and telescopes through which it was ascertained, was to bring the “remote” and “inaccessible” nature closer. The image documented by the previous, truth-to-nature epistemic objectivity, was idealized, perfected, and it was a characteristic specimen but not the exact nature. Mechanical objectivity thus made the perfected nature imperfect and instead aspired to present the object of nature “just as it was” (Daston and Galison 2007: 36, 44–45). In transiting from truth-to-nature to mechanical objectivity, nature became variable, accidental, impure, and unknown – but also real and inaccessible (Daston and Galison 2007: 51).

However, expelling everything that was subjective, and capturing the real and exact nature created anxiety among scientists. It was often expressed that representing the “real” nature in all its complexity was impossible. For example, an anatomist, Albrecht van Haller, complained that “the infinite labor was required to trace the labyrinthine variety of the arteries, which even numerous dissections had failed to coalesce into a clear pattern” (Daston and Galison 2007: 81). How to ensure what was represented was indeed a real representation of nature? Would expelling all subjectivity be enough to ensure it?

What is important to note here is that in selecting the typical and ideal images while pursuing the epistemic objectivity of truth-to-nature, scientists searched for the regularity of “God’s law,” the perfection of which can then be admired. Mechanical objectivity did not provide any such reference point. In fact, in mechanical objectivity, objectivity and its pursuit remained “an elusive goal, a destination always just past the horizon” (Daston and Galison 2007: 189). The impossibility of objectivity was also reflected in the impossibility of expelling all subjectivity. A naturalist, Rudolf Virchow, declared that even after trying for 30 years he was not able to de-subjectivize him entirely (Daston and Galison 2007: 189). These scientists struggled with “incoherent scientific objects” (Daston and Galison 2007: 236) and the frustration of falling short of highest epistemic idea of objectivity was widespread (Daston and Galison 2007: 233). As a result, both objectivity and the scientific self that practiced it were declared as “intrinsically unstable” (Daston and Galison 2007: 250). On the account of this impossibility of ensuring correspondence of their representations to nature as real, “seeing nature as it is,” scientists became, in Daston and Galison’s own words, “well-nigh maniacal” (Daston and Galison 2007: 66). For the truth-to-nature epistemic virtue, the reference point of the perfection of God’s law granted the truth of knowledge. But there is no such reference point available in mechanical objectivity to grant such certainty of knowledge. How did this shift in the attitude towards nature – the mode of modern science – constitute the subject?

It was Alexandre Koyre who argued that the birth of modern science is concomitant with the transformation of philosophical attitude; it marked the reversal of the value attached to intellectual knowledge in comparison to sensible experience. Lacan closely followed Koyre’s conviction that it was Descartes who formulated the principles of new science marking the transformation in philosophical attitude which was attributed to Cartesian *Cogito* in the form of the belief in human rationality. Following Koyre, Lacan said, “For science, the *Cogito* marks the break with every assurance conditioned by intuition” (Lacan 1995 [1964]: 261). Lacan thus dubbed the *Cogito* endowed with capacity for reasoning as “subject of science.”^4^ This, Lacan describes as, “…. a certain moment of the subject that I consider to be an essential correlate of science….the moment Descartes inaugurates that goes by the name *Cogito*” (Lacan 1965: 4). Lacan further states, “…the modification of our subject position….is inaugural [moment] therein” of modern science, but also that “science continues to strengthen it [subject position] ever further” (Lacan 1965: 5).

Lacan’s argument about the subject of science has two more contexts. Firstly, he argues that Descartes’ *cogito ergo sum* is neither about knowledge nor about existence but about certainty. Lacan attributes Descartes’ desire for certainty through the exercise of reason as significantly forming the modern subject. Secondly, Lacan further argues that this subject of modern science is split between knowledge and truth. For Descartes, while distinct observations are prerequisite for the construction of knowledge, it does not grant truth in itself; in other words, knowledge is not inherently true. Descartes invokes God – a non-deceiving agency outside of himself – to guarantee the truth of knowledge. The subject then pursues his own reason in pursuit of knowledge, and God guarantees the truth of this knowledge. Knowledge and truth are therefore separate, in fact truth is external to knowledge, and only God joins them together. But God – the benevolent, good natured God – is an assumption, in fact it is only the faith of the thinking subject (Lacan 1965: 35, 1977 [1964]).^5^ It is the subject who imagines God who then grants truth to knowledge. The subject of science, *Cogito*, therefore is split between knowledge and truth and it is joined together by the (subjective) faith in God as symbolic (and not real) guarantee of truth outside of knowledge.

In discussing truth-to-nature epistemic objectivity followed in the 17^th^ and 18^th^ centuries, Daston and Galison also point out how it was God in whose image the truth or certainty of knowledge was granted. In the pursuit of perfect, pure, average, typical and ideal images in nature, the scientist-subject was searching God’s law that was worthy of admiration (Daston and Galison 2007: 68). The search for the eternal and non-deceiving agency to grant the truth signified the split between knowledge and its truth in the sense that truth was always external to knowledge. Simultaneous pursuit of knowledge and disavowal of its truth thus characterized the subject of science.

However, in modernity, the reference made to God to guarantee truth has significantly been altered. In fact, the God as truth-guarantor (in other words, God as symbolic guarantor), has acquired different names in the secular world. The faith in God is re-articulated and in turn placed in science itself. This faith is placed not just in science but in science’s subjects, in the scientific community’s confidence to deal with complex matters, in mechanical devices, in scientific method such as empiricism, logic, or mathematics. Alternatively, this faith to guarantee truth is entrusted to objects of science such as neurons, genes, or forces of nature (Glynos 2002: 65–66). These objects of science in fact acquire the place of demigod, for instance, the way the gene is made to comprise the immortal essence of humanity in Richard Dawkins’ work on the selfish gene (Dawkins 2006).

In *Science and Truth*, however, Lacan makes the point that the simultaneous pursuit of knowledge and the disavowal of its truth characterizes the subject of modern science. In making the methods and objects of knowledge to provide certainty and truth, modern science has progressively reduced truth to knowledge, in the sense that truth is increasingly located not outside, but inside knowledge. And as a result, Lacan argues, the modern Cartesian subject split between knowledge and truth is erased or sutured. One of the main messages of *Science and Truth* is the way modern science effectively enables the scientist to forget his subjectivity, it actively forgets the subjective drama of its practitioners, “let’s say that [science’s] subject is not often studied” (Lacan 1965: 18). Lacan further argues that the individual consequences of forgetting subjectivity are manifested in mental anguish, suffering, even madness. Lacan counts Cantor (quoted at the beginning of the essay) and Mayer in the list of such “first-rate tragedies” (Lacan 1965: 18).

Although Daston and Galison also arrive at the similar conclusion – in their argument, the demands of objectivity make the scientist-subject to deny his/her subjectivity, and many such scientists expelling their subjectivity in pursuit of representing nature as real became “well-nigh maniacal” (Daston and Galison 2007: 66) – there is a significant difference between theirs and Lacan’s argument. For Daston and Galison, the erasure of subjectivity ensures objectivity, but for Lacanian psychoanalysis erasing or forgetting subjectivity means paranoia.

For Lacan, not just scientific, all knowledge is paranoiac. And still there is a significant difference between the paranoia caused by the mode of modern scientific practice and the foundational aphorism of “all knowledge being paranoiac” in Lacan’s intellectual oeuvre. Paranoia is derived from Greek: para means outside or beside oneself and therefore beyond intelligible thought. For Freud, paranoia is “the blindness of the seeing eyes not wanting to know” (Freud 1893–1895: 117). In other words, what is paranoid is that stands in opposition, alien to the self. It is not just in Lacan, but in the entire Freudian tradition of psychoanalysis, knowledge stands in dialectical relation to fear, the fear of not-knowledge. And, not-knowledge means negation, conflict or suffering that knowledge can cause. “My knowledge started off from paranoiac knowledge,” says Lacan (1988 [1954]: 163) In Lacan’s work, the epistemological context of paranoiac knowledge is situated in three contexts or orders of being – imaginary, symbolic and real. Firstly, knowledge is paranoiac developmentally. It begins with the “mirror stage” because it is acquired through our imaginary relation to the self and the other. In Lacanian psychoanalysis, the “mirror stage” that occurs between 6 to 18 months of infancy is the initial point of self-discovery when the nascent ego or “I” is discovered in the looking glass or through the eyes of another as metaphorical representation of the mirror. “The original specular foundation of the relation to the other…is the first alienation of desire” because it is fundamentally rooted in the imaginary. The ego first recognizes itself in an object outside of itself in the realm of the imaginary, in the illusory order, “in a fictional direction,” and hence the source of alienation and paranoia (Lacan 1988 [1954]: 176). Secondly, the Lacanian subject discovered in imaginary but lost in alienation is further recovered through the Other – the symbolic. “I” is never absolute or autonomous apart from the Other. The Other is “socially elaborated situations” mediated by linguistic structures, “I” is linked to the “Other,” “I” exists as the Other (Lacan 1977 [1936]: 5). However, the Other – to repeat, socially elaborated situations mediated through linguistic structures – especially the Other’s desire, is a potential threat to the subject, because it is an alien force, and hence the source of paranoia (Lacan 1988 [1954]: 185). The third order of being, the “real” is the place of limit, the realm of unconscious, that which is lacking articulation in the imaginary and symbolic orders. The experience of knowing the real is the most horrific because it can never be known in itself. Freud compares the realm of the unconscious with the realm of the nature, “the unconscious is the true physical reality, in its innermost nature it is as much unknown to us as the reality of the external world” (Freud 1900: 613). In Lacan’s work, therefore, all knowledge is paranoiac because the one that emerges from the orders of imaginary and symbolic is loaded with alienation, opposition, and demand. However, this knowledge is paranoiac knowledge not because of the fear of the unknown, but because it is tainted by the fear of knowing a particular truth of the self and the other, this truth that the subject may find horrific. But in the domain of the real that the knowledge becomes impossible, because it acquires indescribable language, this knowledge is paranoiac because it is beyond mind, unknowable, inaccessible. Because this essay engages with the world of the real as represented in modern science, I have not given due space to the paranoiac knowledge of the subject emerging from the domains of the imaginary and symbolic and have focused more centrally on paranoia of the subject of science wanting to know the real.

History of objectivity in Daston and Galison’s detailed work shows how in transiting from practices of truth-to-nature to mechanical objectivity all traces of God or *symbolic* guarantee is replaced with *real* guarantee. The shift from the truth-to-nature to mechanical objectivity is signified by representing nature not as ideal and perfect version that the God’s law has created but as nature really is. Modern science developed increasingly sophisticated experiments and other methods to capture the reality of the real and to establish certainty of knowledge which almost always failed to guarantee such certainty and truth, and in this sense the real almost always remained unknowable. Daston and Galison discuss the way several scientists accounted for this impossibility of representing the real, some examples of which I have already mentioned. The other side or perhaps the precondition of this search for the real is what Lacan said, “there is no Other of the Other” (Lacan 1977 [1964]: 77). In other words, there is no real guarantor behind the symbolic guarantor, first God and then science – God is only a matter of faith and so is science. It means how history of science since the time of Descartes has moved in the direction of foreclosing the symbolic guarantee for the truth of knowledge and replacing it with the real guarantee. As long as the scientific practice tries to find the real behind the symbolic, it will end up with “elaborate” and “grander” theories to bridge the gap, it will end up with proliferation of scientific knowledge and unending displacement of one scientific object for investigation to another. And in this sense the modern scientific practice resembles paranoia, not only because the knowledge of the real is beyond mind, but also because paradoxically this impossibility produces highly elaborate, rigorously logical, but delusional systems of knowledge (Glynos 2002: 65).

Relating this discussion to Daston and Galison’s *Objectivity* would mean that the practice of objectivity emerges from the Cartesian subject’s need for certainty. The paranoia that this need ultimately generates makes the “affective,” the psychological, preceding and driving the epistemological.

### “Pathos, not Ethos Drive Epistemology”

The scientist-self of mechanical objectivity that Daston and Galison portray matches nothing less than the basic principles of Kantian ethics – the categorical imperative (the duty qua willful rejection of subjectivity) of the scientist-self is on the same side as the moral law (ethics of objectivity). In fact, the rational will of the self is the sole possible authority of the moral law – in other words, there is a complete match between the will and the moral law. Not only that the will of the scientist, which includes extreme modification of the self, is something inherently good but the moral law (the ethics of objectivity) is also unconditionally good in itself. In Kantian ethics, the categorical imperative is on the same side of the good (well-being) of the fellow-men. How ethics of objectivity will ensure the good of the people is not clear in *Objectivity.* The same question that is often posed against Kantian ethics can also be raised here: What if duty and good are on the opposite sides? What if duty could be accomplished at the detriment of the fellow-men? In other words, the notion of the good also heralds a pertinent question: Whose good? Daston and Galison, however, are clear on this point. They think that we must first know what objectivity is before we decide if it exists or it is good or bad. This is a fair argument.

To explore this logic further, in Kantian ethics, duty is only that the subject accepts as her duty, it does not exist in an external list of, say, Ten Commandments. The moral law is not some entity that prescribes “do this” or “do that” but it is the law that commands to perform duty without even naming it. In other words, the subject cannot claim that the duty was imposed upon her, that her action only followed the commandment of the law. The subject is responsible for her duty; she cannot hide behind the externally imposed moral law. Otherwise the ethical subject is just an agent of the moral law, an unnecessary and dispensable element of the moral law. Making the duty as one’s own is at the core of the relation between the will and the moral law. To relate this reading to *Objectivity*, Daston and Galison’s argument of the co-implication of objectivity-subjectivity perfectly match with Kantian ethics in the sense that the ethical subject does not bring into a moral situation all subjective and affective baggage (which then need to be removed or expelled) but the scientist-subject is born out of the moral situation – born out of the demands of the objectivity, she emerges from the moral situation of requirements of objectivity, she is made of, she is constitutive of the law of the objectivity. According to Kant, therefore, the subject acts contrary to her well-being and pathological interests only for the reasons of the moral law. In this sense the subject then performs an ethical act, i.e., when she goes against her well-being and pathologies, she is either angelic or diabolical – neither of which could be applied to human beings – and hence Kant says that an ethical act that goes against the well-being and pathologies of the subject is impossible (Zupancic 1998: 59). This impossibility pushes the subject to the manifestation of doubt and guilt and heralds a further indefinite struggle to separate herself from the pathology.

In the critical scholarship on Kantian ethics, it has been a long standing question if Kant thought the reason qua will alone as the impetus for moral motivation or did he think there existed desire, feeling or affect, prior to the will, as source of motivation to moral law. Is reason independently motivating or it requires a priori “affect” for the incentive for motivation? Kant himself wonders how it is possible for the “will without an object of representation” to be the direct incentive for the moral law and concludes that “this is an insoluble problem of the human reason” (Kant 1993: 75). There are scholars who insist that Kant put ethical principles as the absolute source of moral conduct (Nagel 1970: 11, 13), and there are others who think some other motivational factors beyond reason alone are necessary to account for moral obligation (Bond 1983: 11). In the chapter *Incentives of Pure Practical Reasons* in the *Critique of Practical Reason*, Kant meditates on “in what way the moral law becomes an incentive” for the will (Beck 1960: 217). But, here, contrary to his usual position on how moral law motivates human will through pure reason, Kant talks about the effects of the objective moral law on the human subject. This is because the human constitution is made of both a sensuous nature causing incentives and a rational nature which gives moral commands. And in the human being the commands of reason conflicts with the inclination or incentives of sensuous nature. The freedom to act independently of sensuous inclinations results in pain – this, Kant called “negative subjective effects.” These negative subjective effects are pathological in nature because derived from our sensuous nature. The negative subjective effects are not the only source for pathology, though (Beck 1960: 216). Kant also acknowledged that human actions are governed by another law, the law of the faculty of desire. “Life is the faculty of a being by which it acts according to the laws of the faculty of desire. The faculty of desire is the faculty such a being has of causing, through its representation the reality of the objects of these representation” (Kant 1993: 10). The faculty of desire is represented by a certain object, which could be shame, honor, fame, approval of others – on the contrary, the faculty of will is not represented by any such object. And the subject is “affected” by these representations which is the cause of her action. For Kant, when affection is the cause of action, the actions are determined pathologically. Kant also talks about the “positive subjective effect” of the sensuous nature, which he calls “respect” for the moral law, or the moral feeling, which in German he calls *Achtung*. Kant attributes the correspondence of the moral law with the will to the presence of what he calls the “feeling of respect.” The feeling of respect means that the law is nearby; it indicates the presence of the law; it provokes the feelings of fear, admiration, wonder and awe – a feeling of sublimation. Although for Kant, the feeling of respect (for the moral law) “is a singular feeling, reason which cannot be compared with any pathological feeling,” it is the only drive of *pure* practical reason (Kant 1993: 74–75).

This debate over “pathological” component in Kantian ethics continues depending upon which part of Kant’s vast array of intellectual work is invoked. For instance, Zupancic contends how Kant did not see that the feeling of respect could turn into pure and simple, *Ehrfurcht*, i.e., wonder – something that Kant himself linked to fear, thus becoming a perfectly pathological motive (Zupancic 1998: 67). Sytsma, on the other hand, discussing the role of *Achtung* in Kant’s moral theory, interprets how “respect” is produced solely by reason, and thus respect for the law is not the incentive to morality but morality itself and hence far from pathological (Sytsma 1993: 121).

To sum up this discussion, firstly, for Kant a pure ethical act that goes against the well-being and pathologies of the subject is not possible; secondly, what Kant calls pathologies are objects of representation such as shame, honor, fame, approval governed by the faculty of desire; and thirdly, the respect for the moral law is the only pure drive for practical reason.

In this context, many of Daston’s *affect saturated values* would qualify as pathologies in Kantian ethics. In *Objectivity*, Daston and Galison argue that the whole purpose of making atlas within the broad scope of truth-to-nature and mechanical objectivity was to establish standards for the entire disciplinary community for generations to come (Daston and Galison 2007: 202). Atlases were one among many scientific values and practices that meant to bind the dispersed members together and generate the sense of loyalty among them. “Internalized and moralized, these loyalties stamped a distinctly scientific self, which are recognizable across a diverse range of local contexts,” say Daston and Galison (Daston and Galison 2007: 203). To repeat, in Kantian ethics the feeling of respect, the feeling for admiration and awe for the moral law, which in Daston would mean demands of objectivity, is the only pure drive of practical reason. Any other thing, even the affect of loyalty or desire for approval of others would mean pathology.

As I have already discussed, responding to the reviewers, Daston and Galison argued that the notion of *affective states* are the ones in which the psychological and the epistemological, on the one hand, and the individual and the collective, on the other, are made to converge. Daston and Galison do not provide any explanation to the term *affective* neither do they make any gesture in relating it with the massive debates on the *affective turn* in social sciences, body studies, human geography, and media studies. The meaning of the affective is left to the imagination of the reader – and in my interpretation, it is broadly understood as the state that is embodied and deeply emotional but it is not reduced to the purely psychological.

Lorraine Daston in her work has written about what she calls *affect saturated values* – a specific constellation of emotions and values, in a well-defined relation to each other, hanging in balance – upon which science depends for its practices (Daston 1995: 4). Similar to the arguments in *Objectivity*, the moral in the moral economy refers at once to the psychological and to the normative. Daston categorically emphasizes that moral economy is not a matter of individual psychology; she insists, “although the moral economies are about mental states, these are the mental states of collectives”; “it is not a matter of individual psychology”; “this is a psychology at the level of whole culture or subculture” (Daston 1995: 4). The examples of affect-saturated and shared values include devotion to clarity, accuracy, sociability among colleagues, impersonality, impartiality, moral obligation to discipline, diligence, fastidiousness, thoroughness, caution, integrity, trust, civility, curiosity, honesty. Paul White comments that as the model of moral economy of *affects saturated values* was refined in Daston’s work, the affects retreated and morals held sway – “plenty of civility and very little feelings”(White 2009: 796). In the background of the discussion on Kantian ethics, all these collective affect-saturated values making epistemology would qualify as pathological rather than ethical, because in Kant all morality, by its definition, would exclude all pathological motives. To remind the readers, this pathology would include actions caused by the faculty of desire, represented by certain object, for example, loyalty or fame or shame or approval. Although fame and shame are not listed in Daston’s *affect saturated values*, the point I intend to make is that even in Kantian ethics the pure act of reason, in other words, the moral law perfectly corresponding with the will is not possible. This would mean that even in Kantian logic the practices of objectivity are as much pathological as ethical. They are driven by pathos as much as by ethos.

### “Appearance is not Being”

Daston and Galison clarify that they are after understanding a certain “prototypical knower” of nature, a type of scientist as a regulative ideal, as opposed to any flesh and blood individual (Daston and Galison 2007: 204). They are interested in delineating the normative force of historically specified exemplary persona (Daston and Galison 2007: 44). Metaphorically, they prefer “superficiality of enlargement” to “excavating conjectured depth.” Also, their aim is to reveal patterns on how objectivity and subjectivity – two epistemologies, two ethics, two ways of life – intertwine so closely (Daston and Galison 2007: 205).

The enlarged view of the normative persona, however, does not necessarily coincide with the Foucauldian premise on *technologies of the self* that they adopt to explain the activation of such persona. In *Moral Economy of Science*, Daston postulates that the co-production of feeling and seeing happens in science schools by the way of apprenticeship – by making the members of the science inculcating self-discipline and self-surveillance on their own instead of being pushed or coerced. Daston refers here to Foucault’s *technologies of the self* and clarifies that this is an exercise of a microscopic Foucauldian sort of power which structures the way the scientist comes to know (Daston 1995: 6). Daston and Galison emphasize the same point in *Objectivity* and argue that “technologies of the self – the practices of mind and body that mold and maintain a particular kind of self” (Daston and Galison 2007: 198–199). These practices include training the senses in scientific observation. Therefore, keeping lab notebooks and drawing specimens go hand in hand with monitoring one’s own belief and quieting the will (Daston and Galison 2007: 199). As already mentioned, Daston and Galison clearly declare that they are interested in the normative force of regulative ideal and not in the flesh and blood individual. However, in arguing that the normative ideal moulds and shapes the self, and that it compels the scientist-self to quiet the will and monitor the emotions, Daston and Galison take *appearance for being*. They assume that the normative and regulative ideal is perfectly realized in the scientist self being molded and shaped in the processes of apprenticeship.

Based on Lacanian psychoanalysis’ challenge to Foucauldian power and knowledge, I aim to argue below that appearance and being never coincide. Instead, the discordant relation between the appearance and being is not only the condition for desire but it explains the existence of conscience. And the place of the social surface – in terms of Daston and Galison’s enlarged normative view – and the place of desire or conscience or guilt of the flesh and blood historical subject could be in contradiction.

Firstly, I aim to argue that under the panoptic gaze of *technologies of the self* the scientist-self becomes fully visible, governable, and tractable. In fact, the scientist-self becomes visible not only to others but to oneself through the specific, historically constructed norms and standards. However, what is implied in the argument that the scientist-subject is constructed by the panoptical gaze is that the subject thus is constructed by one monolithic discourse. The panoptic argument is ultimately resistant to resistance – in other words, it disallows a subject that can transcend the regime of power.^6^ Also, the panoptic argument ignores the fact that the subject produced by the signifying system can never be determinate. The subject is indeterminable by its articulation that may result under the influence of a multitude of different discourses (Sangren 1995). Secondly, Joan Copjec in her Lacanian critique of Foucauldian historicism argues how the scientist-subject is formed in and by the field of science, but that the subject is never fully formed in this way. She refers to Bachelard to argue that the scientist-subject is split between two modes of thoughts, one governed by historically determined scientific norms, and the other that are eternal, spontaneous and purely mythical (Copjec 1994: 20–21). Copjec further discusses how Bachelard counted the obstacle of imaginary as the reason for only the partial success for the field of science to form the subject. Judith Butler elaborates on this point in her meditation on *Psychic Life of Power* – it was Louis Althusser who further advanced Bachelard’s theory to show that the category of imaginary is not an obstacle, on the contrary, it is absolutely necessary and integral part of the historical process of construction of the subject (Butler 1997: 111–113). Daston and Galison do not account for the role of the imaginary in the historically determined ideological formation of the subject and therefore their argument depends upon the assumed verisimilitude between the ideal, the regulative, the normative and the real referent. Although they have emphatically declared that their inquiry is focused on the normative scientist-subject formed by the field of objectivity and not on the actual social individual, the Foucauldian argument of *technologies of the self* could be understood only with reference to the actual social setting, however hypothetical it might be. In fact, in Althusser, the imaginary, a correction on Bachelard, almost exclusively bears the burden of ideological construction of the subject. Meaning, the imaginary is constitutive of the ideological. On the contrary, the categories of imaginary are barely given any space in Foucault’s, and by their following Daston and Galison’s definition of the normative laws that govern *technologies of the self* (Copjec 1994: 20–24). The law is thus unconditional, it must be obeyed; in fact, *being* by definition is obedience. Hence, although the images of self-surveillance and self-correction are required to construct the subject, if the subject thus constructed is absolutely upright and correct, fully formed, these categories of self-surveillance and self-correction should become redundant. The Foucauldian *technologies of the self* fail to take into consideration the dialectics between the norm and desire and the element of indeterminacy that desire introduces in the making of the subject. And therefore, the enlarged normative view would have made proper sense only if it were juxtaposed with the deeper excavation of how these norms actually work out in the individual scientist life.

### “The Role of Unconscious in the Making of ‘Science as Habit’”

In locating the epistemic fear as an affective state, the book *Objectivity* makes a connection with Daston’s earlier works on *The Moral Economy of Science* (Daston 1995) and with her later work *On Scientific Observation* (Daston 2008). In the latter, Daston reflects on psychology and proposes to bridge the gap between psychology and epistemology – clarifying again that psychology in question was collective rather than individual (Daston 2008: 97). She argues here that learning to see like a scientist is a matter of accumulated experience, a matter of habit formation of a well-trained collective. But acquiring this experience is an equation of time – the scientist has to go through a gradual process of training before experience turns into a habit whence for a mature scientist it becomes possible to see things all-at-once – a naturalist in 1922 described it as a “jizz,” informs Daston. The jizz is an observation that is sure, swift, silent, and happens without a pause for mental analysis (Daston 2008: 101). But before the scientist comes to the position to be able to see things all-at-once, in a jizz, as a matter of habit, she goes through a process of learning in which perception turns into memory and experience. And, Daston clarifies, even when this is all about the production of conscious reason, “the faculty of epistemology has no inkling as to how it’s done” (Daston 2008: 102).

Referring to Ludwik Fleck’s G*enesis and Development of a Scientific Fact*, Daston further emphasizes the trope of temporality and argues that this knowledge is not tacit, retroactively it is perfectly possible to describe this process of habit formation – the way perceptions coalesce into experience – in considerable detail, in a series of stages and thus bring it to the modes of representation. However, while in action – while the scientist is seeing things all-at-once, in a jizz – the conscious reason and faculty of epistemology has no idea how this is done. Daston here refers to Ludwik Fleck’s interpretation of thought collective and also genesis of the scientific fact. Fleck describes the process of genesis of the fact – it arises after “a signal of resistance in the chaotic initial thinking, then a definite thought constraint, and finally a form [of the fact] to be directly perceived” (Fleck 1981 [1935]: 95). The genesis/crystallisation of the fact in a thought collective is described in a musical metaphor – “the confused notes followed by hummed and inaudible tunes gradually turning into a melody once the “co-workers” listened and tuned their sets until these became selective” (Fleck 1981 [1935]: 95). The melody could then be heard even by the unbiased person – meaning, it crystallises into a fact. The genesis of the scientific fact thus is not only a process in the sense that it is emergent through time, but most importantly, it involves a “chain of experiences” – first chaos, then constraint, then perception and then fact emerges. There is similarity between Daston’s experienced scientist able to see in a jizz, all-at-once, and Fleck’s genesis of the scientist fact – both are processes, are emergent, subjected to time, and both constitute a chain of experiences that is although silent and swift in Daston, but can be retroactively traced into a series of stages in both Daston and Fleck.

However, there are subtle ways in which the irrational and rational, reasonable and unreasonable, conscious and unconscious, feelings and thought, experience and fact are intertwined in these accounts. In *Objectivity*, Daston and Galison refuse to call “epistemic fear” anything than epistemic, because it could otherwise open the door for murky realms of the “irrational”; the historians of science are very wary of these – Daston informs us (Daston 2008: 101). Here, the individual subjectivity, a specific emotional trajectory, the psychology of innermost thoughts, and other affective intentions of historical actors are relegated to mind reading, and to biographies of the individual scientist. For Daston, the epistemic fear is structural and collective and hence “there is nothing irrational (or reductive) about [it],” in fact, “it is simultaneously reasonable, psychological and collective” (Daston and Galison 2008: 672). In my reading, it is Daston and Galison’s own fear of “irrationality” that makes them fear the epistemic fear for being anything else than epistemic.

But then, even after arguing to make the epistemic fear “not irrational,” Daston still has to enter in the murky ground in which conscious and unconscious and thought and feeling are inseparably intertwined – for instance, the jizz happens without a pose for a mental analysis, here the emergence of the conscious scientific reason has to go through the path of the unconscious; the way perception turns into memory into experience into habit has a psychology. And yet again, the conscious reason, the faculty of epistemology, have no inkling how it is done when it is done (to repeat, the stages of this process can be retroactively traced, though). This all-at-once-ness in Daston is explained in the words of Descartes, “the arguments so speeded up that it bursts upon the mind as a single cognitive event” and “[n]o amount of explicit reasoning, even mathematical reasoning, can compete with it” (Daston 2008: 110).

In these arguments of Daston the meaning of the psychological finally arrives home. The jizz, all-at-once-ness, is not about some *random* eruption of unconscious upon the conscious. This is about the unconscious fundamentally structuring the conscious reasoning without the conscious even knowing it. In fact, this is about the *indispensability* of the unconscious in ordering the conscious. The claims of all-at-once-ness, the jizz, the arguments bursting upon the mind as a single cognitive event, “they concern,” Daston writes, “largely unconscious processes of perception” happening “albeit processes that are consciously…. taught and controlled by the exercise of scientific observation” (Daston 2008: 105). And still, instead of accepting this crucial role of the unconscious in structuring the conscious, Daston’s tone turns defensive, she insists that even when the perceptual habit – the jizz and all-at-once-ness – “is not *of* reason,” or more than reason, “this does not render such habits *ipso facto* irrational” (Daston 2008: 105). She repeatedly asserts that, “[t]here is nothing individual, nothing arbitrary, nothing mystical, [nothing irrational] about this kind of psychology” (Daston 2008: 106). It’s the fear of epistemic fear lapsing into individual and hence subjective and hence irrational that drives Daston and Galison to claim that the fear driving epistemology is purely and only epistemic. I can’t but mention here that Fleck, whom Daston significantly refers to make her point, however, is upfront in accepting the “irrationality” of “being experienced.” He says, “[t]he ability directly to perceive…. is acquired only after much experience….The concept of being experienced, with its hidden *irrationality*, acquires fundamental epistemological importance” (italics mine) (Fleck 1981 [1935]: 92). My claim is that Daston’s conceptualization of the terms like unconscious, psychological, individual and collective are underdetermined because incorporating their meaning in all richness will threaten the master-self of Daston and Galison that is made of the Kantian “ethical imperative” at the core. As already discussed at length, Daston and Galison insist that “the link between epistemology and ethics” is very strong. Whatever the epistemic virtue, “the exhortations are nearly always religious and ascetic in tone. There is always an ethical imperative at core“ (Daston and Galison 2007: 40).

The role of the unconscious is truncated in Daston’s work in the degree to what is needed to aid what Fleck calls the “chain of experiences” coalescing into “the jizz,” and “the all-at-once-ness.” In other words, the unconscious is there only to silently and swiftly structure the experienced scientist’s epistemic conscious. Daston here employs the concept of the unconscious in a selective manner without referring to its proper origin either in the tradition of Freudian psychoanalysis or even prior to that tradition. The incorporation of the meaning of the unconscious as it is understood in the Freudian tradition would seriously jeopardize the self-mastery at the center of the practices of objectivity in Daston and Galison’s *Objectivity*. The Freudian subject is fundamentally split between the conscious and unconscious. In fact, the unconscious does not come to rescue, aid, or structure the agency of the conscious, but its repressed drives put serious obstacles in the path of the rational conscious, and these drives are obstinately resistant to control and even comprehension by the conscious. The agency of the unconscious, as it is projected in Daston, is something akin to “psychological unconscious” and not the genuinely Freudian unconscious, which, Felicity Callard, discussing such debates in human geography, argues, constructs an idealized psyche because it is far easier to deal with it and to make it susceptible to change and transformation, it is an accessible psyche, conducive to agency, and hence available for political maneuvering. But this is not the way the psyche necessarily operates if the psychoanalytical concept of unconscious is genuinely taken into consideration (Callard 2003: 308). And if the Freudian notion of the unconscious is taken into consideration, the Kantian notion of self-mastery at the core of objective knowledge production will come under serious challenge.

## Conclusion

The main focus of this essay is to critique the neo-Kantian scientist-self in Lorraine Daston and Peter Galison’s book *Objectivity*. While doing so, I have also closely engaged with Daston’s later and earlier work *On Scientific Observation* and *The Moral Economy of Science.*

The essay challenges Lorraine Daston and Peter Galison’s formulation of the neo-Kantian self at the core of the scientific-subjectivity. They posit that the scientist-self is first and foremost normative, it is determined to do the right thing, i.e., fulfill the ethos of objectivity. In other words, for Daston and Galison the “categorical imperative” (the duty qua willful rejection of subjectivity) of the scientist-self is on the same side as the moral law (ethics of objectivity). Based on this conception of the master-self, Daston and Galison argue that the link between epistemology and ethics is so strong that whatever the epistemic virtue, there is always ethical imperative at the core. This scientist-self is affective (fearful) only in the noble pursuit of objectivity. In fact, the fear of subjectivity is the fear that the core self would become an obstacle in the demands of objectivity and hence this fear is foremost ethical and normative. Daston and Galison’s scientist-self is thus made of active self-consciousness upon which both the ethics of knowledge and knowledge are built.

I have presented four challenges to Daston and Galison’s central argument that epistemology and ethos fuse. Firstly, following Jacques Lacan’s work, I have argued that the subject of science constituted by the mode of modern science suffers from paranoia. It is not the fear of subjectivity interfering with objectivity but the impossibility of knowing the truth of the real (of the nature) that causes paranoia. This paranoia, on the one hand, caused by the erasure of subjectivity triggers much suffering in the scientist-self, and on the other, creates grander and elaborate scientific theories that almost always fail to provide the truth or certainty of knowledge. Here, I have argued that it is not ethos of objectivity that drives epistemology but pathos of paranoia. The second challenge builds upon Kant’s own denial that the perfect correspondence between the human will and the moral law is possible. Kant himself thought that an ethical human act is impossible without the component of pathology. Thirdly, referring to the contestation of historicism in Foucault’s work from the location of the existence of desire as the formative part of subjectivity, I posit that Daston and Galison take appearance for being. Daston and Galison have argued in *Objectivity* that they are concerned only with the prototypical knower, what they call the enlarged view of the normative persona, and not with the experiences of flesh and blood individuals in real historical and cultural settings. They, thereby, assume verisimilitude between the ideal, the regulative, and the normative and the real referent. In brief, the problematic of the indeterminate and often contradictory relationship between the normative and the real is pointed out. The fourth challenge questions the notion of psychological and unconscious in the making of epistemology in Daston’s earlier work on *The Moral Economy of Science* and *On Scientific Observation*, which also make close connection with Ludwik Fleck’s *Genesis and Development of a Scientific Fact.* These challenges question, on the one hand, the neo-Kantian conception of the scientist subject, and, on the other, contest Daston and Galison’s argument that ethos and not pathos drives the imperative of epistemology.

Based on these challenges to Daston and Galison’s formulation of the co-construction of psychology/subjectivity and epistemology/objectivity, I wish to claim, following Michael Polanyi (following Sartre), that *existence precedes essence*, or in other words, psychology precedes epistemology. This essay aims to make a case for the argument that understanding and disclosing “entities” in scientific domain presupposes an understanding of “being” in general; it also aims to make a case for an alternative conception of the scientist-subject and correspondingly an affective and existential conception of science that I have further explored elsewhere (Shah 2013, 2016).

## Footnotes

1 As quoted in Burgoyne (2002: 237).

2 Hermann similarly proposed parallels between mathematical and psychic structures in the work of Bertrand Russell (Burgoyne 2002, endnote 25, p. 255).

3 Finding out *how we live as we do now* Steven Shapin explores the relationship between the authority of knowledge and the character of knowers (Shapin 1988, 1991, 2007). More so, the co-dependence of the cognitive and the emotional is also approached by the growing field of existentially oriented scientific biographies. Here, science is projected as existential choice made by scientists (Nye 2006). See also (Soderqvist 1996: 69). Lastly, the oldest of the inquiries on science and the self is made by the feminists. Do women scientists practice science differently than men? Would science be less reductionist, more empathic and intuitive if women had an equal role in shaping it? For a long time, feminist epistemologies have been engaged with theorizing the relationship between objectivity and subjectivity, in which the idea of what can be counted as objective science and who is the knowing subject have been revised from their positivist and foundationalist predecessors. See, for example, Harding (1986, 1987, 1994, 1996). Also see Longino (1993, 2001). For a detailed discussion on feminist epistemologies and their construction of the scientist-subject, see Shah (2013).

4 Dany Nobus attempts to “reconstruct and clarify Lacan’s argument” in the text *Science and Truth.* Nobus clarifies that the expression of subject of science in this text is extremely ambiguous. It simultaneously refers to “the scientist, the topic of study within scientific practice, science itself, the subjective elements within science, and the objects subjugated to scientific investigation.” I also find *Science and Truth* often beyond comprehension. I thereby have closely consulted Nobus’s interpretation that subject of science juxtaposed with *Cogito* in the text means human rationality, mental power, and the certainty of continuous experience of thought (Nobus 2002: 94).

5 Lacan’s *Science and Truth*, the transcript of the opening seminar on *The Object of Psychoanalysis*, was first published in 1965 (Lacan 1965). Many ideas in *Science and Truth* emerged in Lacan’s previous seminar on *The Four Fundamental Concepts of Psychoanalysis* which were subsequently summarized in the paper *Position of the Unconscious*. In this trail of thoughts emerging over a year, Lacan was concerned with the subject of science and the scientificity of psychoanalysis but also about the way unconscious was structured like language. Many scholars, including those who are expert on Lacan’s oeuvre have complained that the transcribed and translated text of *Science and Truth* is fairly unintelligible and inaccessible. It is crowded with a multiplicity of ideas presented in highly fragmentary ways. Lacan in general is a difficult philosopher to follow. For example, Jon Mills, one of the prolific commentators of Lacan’s work, describes his writing as fragmentary, chaotic, incoherent, his jargon highly esoteric which resists articulate systematization (Mills 2003). Most of Lacan’s writings were originally not written, they were transcribed from lectures and seminars that add an additional layer of inaccessibility, not to even mention the problems created by the translation from French to English. One translator and a long time critical scholar of Lacan’s work, Bruce Fink, put it in the preface of his translation, “Lacan’s French is….so polyvalent and ambiguous that some frame must be imposed to make sense of it whatsoever” (Miller 1975). *Science and Truth* has made sense to me in consultation with other interpretative contributions on “reconstruction and clarification of Lacan’s argument” (Nobus 2002: 89). I have therefore referred to a few such efforts at “reconstruction and clarification” of Lacan’s arguments in *Science and Truth* (Burgoyne 2002; Glynos 2002; Miller 2002; Nobus 2002; Stavrakakis 1999).

6 For the further discussion on resistance in Foucault’s notion of power, see Sangren (1995).
